# Antibody-Mediated Internalization of Infectious HIV-1 Virions Differs among Antibody Isotypes and Subclasses

**DOI:** 10.1371/journal.ppat.1005817

**Published:** 2016-08-31

**Authors:** Matthew Zirui Tay, Pinghuang Liu, LaTonya D. Williams, Michael D McRaven, Sheetal Sawant, Thaddeus C Gurley, Thomas T. Xu, S. Moses Dennison, Hua-Xin Liao, Agnès-Laurence Chenine, S. Munir Alam, M. Anthony Moody, Thomas J. Hope, Barton F. Haynes, Georgia D. Tomaras

**Affiliations:** 1 Duke Human Vaccine Institute, Duke University, Durham, North Carolina, United States of America; 2 Department of Molecular Genetics and Microbiology, Duke University, Durham, North Carolina, United States of America; 3 Harbin Veterinary Research Institute (HVRI), Chinese Academy of Agricultural Sciences (CAAS), Harbin, Heilongjiang, China; 4 Department of Cell and Molecular Biology, Feinberg School of Medicine, Northwestern University, Chicago, Illinois, United States of America; 5 Department of Medicine, Duke University, Durham, North Carolina, United States of America; 6 US Military HIV-1 Research Program, Rockville, Maryland, United States of America; 7 Department of Pediatrics, Duke University, Durham, North Carolina, United States of America; 8 Department of Immunology, Duke University, Durham, North Carolina, United States of America; 9 Department of Surgery, Duke University, Durham, North Carolina, United States of America; Vaccine Research Center, UNITED STATES

## Abstract

Emerging data support a role for antibody Fc-mediated antiviral activity in vaccine efficacy and in the control of HIV-1 replication by broadly neutralizing antibodies. Antibody-mediated virus internalization is an Fc-mediated function that may act at the portal of entry whereby effector cells may be triggered by pre-existing antibodies to prevent HIV-1 acquisition. Understanding the capacity of HIV-1 antibodies in mediating internalization of HIV-1 virions by primary monocytes is critical to understanding their full antiviral potency. Antibody isotypes/subclasses differ in functional profile, with consequences for their antiviral activity. For instance, in the RV144 vaccine trial that achieved partial efficacy, Env IgA correlated with increased risk of HIV-1 infection (i.e. decreased vaccine efficacy), whereas V1-V2 IgG3 correlated with decreased risk of HIV-1 infection (i.e. increased vaccine efficacy). Thus, understanding the different functional attributes of HIV-1 specific IgG1, IgG3 and IgA antibodies will help define the mechanisms of immune protection. Here, we utilized an *in vitro* flow cytometric method utilizing primary monocytes as phagocytes and infectious HIV-1 virions as targets to determine the capacity of Env IgA (IgA1, IgA2), IgG1 and IgG3 antibodies to mediate HIV-1 infectious virion internalization. Importantly, both broadly neutralizing antibodies (*i*.*e*. PG9, 2G12, CH31, VRC01 IgG) and non-broadly neutralizing antibodies (*i*.*e*. 7B2 mAb, mucosal HIV-1+ IgG) mediated internalization of HIV-1 virions. Furthermore, we found that Env IgG3 of multiple specificities (*i*.*e*. CD4bs, V1-V2 and gp41) mediated increased infectious virion internalization over Env IgG1 of the same specificity, while Env IgA mediated decreased infectious virion internalization compared to IgG1. These data demonstrate that antibody-mediated internalization of HIV-1 virions depends on antibody specificity and isotype. Evaluation of the phagocytic potency of vaccine-induced antibodies and therapeutic antibodies will enable a better understanding of their capacity to prevent and/or control HIV-1 infection *in vivo*.

## Author Summary

Emerging data highlight the role of antibody Fc effector functions as immunological mechanisms involved in vaccine and passive immunotherapy efficacy. One such Fc effector function is antibody-mediated virion internalization, where antibodies recognize a virus and engage Fc receptors on phagocytes, causing them to internalize the virus. Although potentially critical for protection from HIV-1 acquisition, the ability of HIV-1 specific antibodies to mediate virion internalization of infectious HIV-1 particles is unknown. We demonstrate that antibodies with different paratopes, isotypes and subclasses mediate HIV-1 virion internalization, using novel HIV-1 internalization assays. Env IgG3 mediated greater virion internalization activity than IgG1, followed by IgA1 and IgA2. Given that Env IgG3 correlated with decreased risk of HIV-1 infection in the one partially efficacious HIV-1 vaccine trial to date (RV144), determining the underlying antiviral mechanisms is critical for improving HIV-1 prevention strategies. Our study provides direct evidence of a new antiviral mechanism against HIV-1 infection, IgG3 mediated virion internalization, and raises the hypothesis that a mechanism of protection mediated by IgG3 could be this improved Fc-mediated antiviral function. These findings have important implications for harnessing antibody effector functions for HIV-1 vaccine design, HIV-1 cure and passive immunotherapy for HIV-1 clearance at the portal of entry.

## Introduction

Antibodies are a critical part of the immune response against pathogens, and exert their protective functions via a multitude of mechanisms that involve both the Fv and Fc domains of the antibody. These include direct pathogen capture and neutralization, as well as mechanisms such as antibody-dependent cell cytotoxicity (ADCC) and antibody-dependent phagocytosis (ADCP) that engage other innate immune cells as effectors to clear infected host cells, immune complexes, and opsonized virus [1]. Apart from direct sequestration and destruction of pathogens, engagement of innate immune cells also influences the downstream adaptive immune response by stimulating the secretion of inflammatory mediators [2].

Recent data highlight that protection against HIV-1 infection as well as inhibition of HIV-1 replication after establishment of infection may be mediated not only by direct neutralization, but also by Fc-mediated antibody effector functions [3–12]. An immune correlates analysis of the partially efficacious RV144 vaccine trial identified that V1-V2 IgG antibodies correlated with decreased risk of HIV-1 infection [3, 4, 6, 13]. These V1-V2 antibodies were not broadly neutralizing but were capable of multiple antiviral functions, such as ADCC, virion capture, and tier-1 neutralization [14–16]. Notably, the RV144 vaccine regimen elicited antibodies that were non-broadly neutralizing but that exhibited coordinated Fc-mediated effector responses [5, 6]. FcR polymorphisms also influenced RV144 vaccine efficacy [17]. Other HIV-1 vaccine efficacy trials that showed no efficacy either lacked a coordinated Fc-mediated effector response [5] or lacked evidence of strong Fc-mediated antibody functions [18, 19]. In rhesus macaque challenge models, non-broadly neutralizing antibody functions, including phagocytosis, correlated with vaccine protection [8–10]. Thus, the results from human and non-human primate HIV-1 vaccine clinical trials raise the hypothesis that Fc-mediated antibody effector functions are an achievable and potentially protective antiviral immune response to induce by preventative vaccines.

Fc effector functions are important not only for vaccination, but also for cure and passive immunotherapy strategies revolving around the delivery of broadly neutralizing antibodies (bnAbs). Although broadly neutralizing antibodies (bnAbs) are defined based on their ability to neutralize a broad range of viruses, recent passive immunization trials show that their protective activity is not solely due to neutralization, but also in part due to Fc-mediated function. In non-human primate (NHP) passive immunization studies, with both high and low dose vaginal challenge of rhesus macaques with SHIV162p3, protection decreased by about 50% when the administered passive antibody was incapable of binding Fc receptors [20, 21]. Similarly, in murine passive immunization studies, antibodies with enhanced ability to bind activating Fc receptors gave greater protection than their epitope-matched counterparts [22]. These findings have also been demonstrated for other viral pathogens. For instance, broadly neutralizing antibodies against influenza demonstrate a dependence on Fc-FcR interaction to mediate protection *in vivo* [23]. A nonfucosylated glycovariant of the anti-RSV IgG, Palivizumab also showed significantly improved protection *in vivo* [24].

In this study, we focus on the antibody Fc effector function of phagocytosis. Antibody-dependent phagocytosis is best known for its essential role in defense against extracellular bacterial and fungal pathogens, but has also been shown to play important roles in clearing viral intracellular infections, including influenza [25–31], West Nile Virus [32], adenovirus [33], SARS coronavirus (SARS-CoV) [34], and foot-and-mouth disease virus (FMDV) [35, 36]. Notably, for both SARS-CoV and FMDV, protection is mediated not by neutralization but by antibody-dependent phagocytosis despite the presence of neutralizing antibodies [34–36]. Further, in FMDV, antibodies mediating antibody-dependent internalization show greater breadth of activity against heterologous strains compared with neutralizing antibodies [36]. In the HIV-1 field, antibody-mediated phagocytosis correlated with reduced risk of infection in NHP vaccine studies and in humans was associated with an IgG3 response that correlated with decreased risk of infection [5, 8, 10]. Since phagocytes are present at the mucosal surfaces that are the sites of transmission for HIV [37], antibody-dependent phagocytosis may play a role in preventing mucosal HIV-1 transmission. A role for phagocytosis in influencing disease progression has also been demonstrated. Polymorphisms in FcγRIIa, which is one of the major receptors responsible for IgG-mediated ADCP [38, 39], correlated with HIV-1 progression and susceptibility [40]. In addition, impaired phagocytosis is one of the hallmarks of chronic HIV-1 infection [41–43].

Different antibody isotypes and subclasses appear to vary in their ability to protect against HIV-1 infection, and one key question in HIV-1 vaccine design is which antibody isotypes/ subclasses should be induced by vaccines to maximize protection. For instance, in the RV144 vaccine trial, serum Env IgA correlated with increased risk of HIV-1 infection [3]. This was potentially due to monomeric circulating IgA blocking IgG mediated ADCC by Natural Killer (NK) cells [44, 45]. Also, V1-V2 IgG3 antibodies correlated with decreased risk of HIV-1 infection. These IgG3 antibodies were associated with Fc mediated antiviral activity by ADCC [4] and phagocytic activity [5], though it is unclear whether the IgG3 profile directly contributed to antiviral activity [6]. Differences between IgG1, IgA1 and IgA2 have also been found for various other effector functions including neutralization, virus capture, and transcytosis inhibition [46, 47]. Differences in antibody physiological localization may also play a role—HIV-1 infection occurs primarily via the mucosal routes, where IgA can be present in higher concentrations than IgG. Thus, the mechanisms behind how antibody isotypes/subclasses affect protective efficacy remain unclear, and require further study. Given that each FcR has varying affinities for each immunoglobulin subclass Fc domain [48, 49], and that Fc-FcR affinity has been found to correlate with phagocytic activity [39], phagocytic activity is likely to vary depending on the FcR and antibody subclass involved. Detailed evaluation of the Fc-mediated antibody function of different specificities and forms of vaccine-induced antibodies and passively administered broadly neutralizing antibodies will improve strategies aimed to prevent and/or control HIV-1 infection *in vivo*. Although the potential protection of antibody-mediated phagocytosis in HIV-1 infection has been discussed previously in several studies, the impact of antibody structure, including both paratope and isotype/subclass, on phagocytosis potency has not been directly assessed. Further, these studies used model systems comprising HIV-1 antigen conjugated beads and monocytic cell lines instead of infectious virions and primary human phagocytes [5, 6, 8–10, 12, 37, 39, 50, 51]. In this study, we assessed the role of antibody in mediating phagocytosis of infectious HIV-1 virions in primary monocytes. We found that antibody-mediated internalization of HIV-1 virions does not require neutralization but is a function of both antibody paratope and isotype/subclass. These findings raise the hypothesis that antibody isotype/subclass profiles may differ in their protective efficacy due to differing potencies in antibody-mediated phagocytosis, which has implications for current vaccination and passive immunization strategies.

## Methods

### Ethics Statement

Human peripheral blood mononuclear cells from HIV-1 negative individuals and vaginal wecks from HIV-1 positive women were collected with IRB approval by the Duke Medicine Institutional Review Board for Clinical Investigations. All subjects were consented following 45 CFR 46 and written informed consent was obtained by all participants. No minors were recruited into this study.

### Primary Monocytes

Human peripheral blood mononuclear cells from HIV-1 negative individuals were collected and mucosal wecks were collected from HIV-1 seropositive individuals. Additionally, monocytes were purified from blood packs purchased from the blood bank (Red Cross). Blood derived monocytes were isolated from peripheral blood mononuclear cells (PBMCs) of HIV-1 negative healthy donors using the Human Monocytes Isolation Kit II by autoMACs magnetic negative selection beads (Miltenyi Biotech), or via elutriation.

### Cell Lines

THP-1 monocytic cells were purchased from ATCC and grown in supplemented RPMI-1640 (10% FBS, 1% Penicillin/Streptomycin).

### Fluorescently Labelled HIV-1 Virions

Fluorescently labelled HIV-1_BaL_-Tomato, HIV-1_92TH023_-Tomato, and HIV-1_CM235_-mcherry were generated as described in [52, 53]. Briefly, labelled virus was generated by co-transfecting 293T cells with a HIV-1 proviral plasmid with a plasmid encoding Gag protein fused with a C-terminal fluorescent molecule and virus stocks were purified from cell supernatant. HIV-1_BaL_ proviral plasmid comprised the R8BaL coding sequence cloned into a NL4-3 backbone [54], while HIV-1_92Th023_ proviral plasmid comprised the 92Th023 gp160 coding sequence cloned into a CRF01_AE backbone.

### Fluorescently Labelled HIV-1 Envelope Antigen Coated Beads

Biotinylated HIV-1 Envelope antigen was conjugated to Neutravidin fluorescent beads (Invitrogen) as described [50]. Briefly, 10 μl of 0.1% BSA/PBS-washed beads were incubated with 20 μg biotinylated antigen overnight at 4°C on a rotator. Unbound antigen was removed by washing twice with 1 ml 0.1% BSA/PBS.

### Monoclonal Antibodies

Epitope-matched subclass-specific recombinant monoclonal antibodies targeting multiple regions of the HIV-1 Envelope (CD4 binding site (CD4bs) (CH31) [55], V1V2 (HG107) [14], gp41 immunodominant region (7B2) [7], and the gp120 CD4 binding site core (CH27, CH28) [56]) and to influenza hemagglutinin (HA) (CH65) [57] were generated from IgG1 and IgG3 gene expression constructs. Briefly, HIV-1-specific immunoglobulin variable heavy-chain (V_H_) and light-chain (V_L_) gene segments were *de novo* synthesized (GenScript) and cloned into a pcDNA3.1 vector containing full length IgG1, IgG3, IgA1, or IgA2 constant region genes or light chain constant region genes and transiently transfected into 293F cells using polyethyleneimine (PEI, Polysciences Inc.). Supernatants were harvested after 4–5 days of incubation at 37°C and 8% CO_2_, concentrated, and affinity purified by protein G or peptide M chromatography per manufacturer’s instructions (Pierce, ThermoFisher Scientific). Antibody purity was evaluated by SDS/PAGE and Coomassie Blue staining for heavy and light chain bands of the appropriate size. Two forms of recombinant IgG1 mAbs were produced: wild-type IgG1 antibodies designated IgG_SEK and the other termed IgG1_AAA or IgG1_4A antibodies, optimized for human FcγRIII binding via introduction of alanine mutations in the IgG1 Fc region at positions 298, 333, 334, and 429 (AAA—S298A/E333A/K334A; 4A –S298A/E333A/K334A/N429A) [58, 59]. Wild-type IgG3 antibodies were produced based on the IgG3 heavy chain constant region coding sequence M12958_X03604.

### THP-1 and Monocyte Phagocytosis Assay of HIV-Specific Antibodies

The antibody-mediated phagocytosis assay was performed as described [50], with the following modifications. Briefly, 9×10^5^ beads (equivalent of 0.1 μl of supplied suspension) or 10 μl diluted fluorescence labelled HIV-1_CM235_, HIV-1_92TH023_, or HIV-1_BaL_ (containing 4.4 ng to 495 ng p24) were mixed with 10 μl (25 μg/ml final concentration) monoclonal antibodies in a 96 well round bottom plate. Each experiment, with positive and negative controls, was performed with the same stock concentration of virus. After incubation at 37°C for 2 hours, 5 x10^4^ (THP-1) or 6 x10^4^ (primary monocytes) of CD4-blocked cells were added to each well with final volume 40 to 200 μl each, then spinoculated at 1200 g for 1 hour at 4°C. CD4 blocking was performed in order to reduce background levels of virus internalization due to Env-CD4 interactions, and blocking was achieved by pre-treating the cells at 10 x10^6^ cells/ml with 20 μg/ml anti-human CD4 antibody (clone SK3) (Biolegend) for 15 minutes at 4°C before adding them to the antibody-beads/virus mixture. Nevertheless, similar patterns of antibody-dependent signals were observed when sCD4 blocking was omitted from the experimental setup. Following spinoculation, antigens/viruses and cells were incubated at 37°C for 1 hour for phagocytosis/virion internalization. After incubation, supernatant was removed and cells were washed with PBS. Cells were then fixed in 2% paraformaldehyde. To calculate phagocytosis scores for both the bead based phagocytosis assay and the virion internalization assay, a cutoff was first assigned based on the 95^th^ percentile of the no-antibody control. For each sample, the % of cells above this cutoff was multiplied by their mean fluorescence intensity (MFI), and then normalized to the corresponding result for the no-antibody control to give the final score. A background level of phagocytosis was determined based on the mean + 3 standard deviations (SD) of non-HIV-specific antibodies. To confirm the robustness of the calculated scores, alternative methods of calculation were also used, including dividing the sample MFI by the no-antibody control MFI without assigning a positivity cutoff, as well as subtracting the sample MFI by the no-antibody control MFI. These alternate methods of calculation gave similar results. Robustness of the assay was confirmed by multiple scientists obtaining similar results with control antibodies and also replicating key phagocytosis experiments. We have obtained a range of phagocytosis scores between 2 to 20, giving a 10-fold assay dynamic range. Where relevant, fold differences in phagocytosis score are calculated by a division of the antibody-dependent internalization components of each phagocytosis score (baseline-subtracted), using the formula (Phagocytosis Score A—1) / (Phagocytosis Score B—1). This calculation is used only when both conditions have an antibody-dependent component.

### Phenotyping FcR on THP-1 and Monocytes

1 x10^6^ THP-1 cells were incubated with Aqua-dye and surface stained with titrated amounts of CD16-FITC, CD89-PE, CD14-PE Cy5, CD64-PE Cy7, CD32-APC, and CD3-Alexa Fluor 700 (BD Biosciences and Invitrogen). Following wash, cells were fixed with 1% formaldehyde. Flow data were acquired on a LSRII flow cytometer (BD Immunocytometry Systems) and the data were analysed using FlowJo software (TreeStar).

### Fc Receptor Blocking Experiments

Receptor blocking antibody mouse anti-human CD89 monoclonal antibody (MIP8a) (Abcam) was pre-incubated with primary monocytes at 0, 1, 5, or 25 μg/ml at 4°C for at least 1.5 hours prior to being mixed with opsonized beads. Percentage blocking is calculated based on a division of the antibody-dependent internalization components of each phagocytosis score (baseline-subtracted), using the formula [1 − (Phagocytosis Score with blocking—1) / (Phagocytosis Score without blocking—1)] * 100.

### Biolayer Interferometry (BLI)

The binding kinetics of CH31 IgG1 and IgG3 were measured on an Octet RED384 (Fortébio). The instrument was controlled by Data Acquisition 9.0 software (Fortébio). Two sets of 8 biosensors were regenerated in glycine buffer pH 2.0 (GE Healthcare Science) for a total of 30 seconds. Then, CH31 IgG1 or IgG3 and the respective CH65 IgG1 or IgG3 at 10 μg/mL were separately loaded on a set of 8 Anti-Human IgG Fc Capture (AHC) Biosensors (Fortébio), with termination of loading upon reaching a 1.2nm shift for each antibody. Matched sets of CH31 and CH65 loaded biosensors were then dipped simultaneously in PBS buffer for 60 seconds to equilibrate the biosensors, followed by 60 seconds in PBS to establish a baseline, followed by 600 seconds in the sample analyte (HIV-1_92Th023_ gDneg gp120, purified by high performance liquid chromatography (HPLC) to obtain monomers) for association, and finally 600 seconds in the baseline PBS for dissociation. Association and dissociation was measured for a range of antigen (HIV-1_92Th023_ gDneg gp120) concentrations, from 3.125 to 200 μg/mL in 2-fold increments, as well as blank (PBS). The temperature, agitation speed, distance of the tip and acquisition rate of the instrument was set at 29°C, 1000 rpm, 4mm, and 5.0 Hz (averaging by 20), respectively. Data fitting with the 1:1 Langmuir fitting model was performed by Data Analysis 9.0 (CFR11) software (Fortébio). Reported association and dissociation values were calculated from curves fitted with subtraction of negative control CH65 IgG1_SEK and CH65 IgG3 from CH31 IgG1_SEK and CH31 IgG3 respectively to exclude non-specific binding interactions. Similar results were obtained for association and dissociation without negative control subtraction.

### ImageStream Cytometry

For ImageStream cytometry analysis of virion internalization, the phagocytosis assay was run as described above, except that a larger pool of cells (5 x10^5^ primary monocytes) at a final concentration of 10 million cells/ml was used. After fixation, cells were washed and resuspended in 1% BSA/PBS. Antibody-mediated virion internalization by monocytes was analysed using an ImageStream^X^ Mark II Imaging Flow Cytometer (EMD Millipore). Fluorescent virus images were collected in channel 4 (595–642 nm) at 40x magnification. Focused, single cells (based on gating on Gradient RMS, Area, and Aspect Ratio of the brightfield image) were chosen for analysis.

### Infectious Virion Capture Assay (IVCA) (Column)

The IVCA method utilizes a Protein G column based capture of Ig-virion immune complexes with two readouts for quantifying total virus particles (RT-PCR) or the infectious virions (TZM-bl infectivity assay) as previously described [16]. Briefly, IgG was mixed with HIV-1 stock at final concentration of 10 μg/ml (200 μl volume) to form Ab-virion immune complexes (IC), which were passed through a protein G column. The infectivity of the flow-through was measured by a TZM-bl infection assay. The total virus particles in the flow-through and the column-captured fraction were measured by HIV-1 *gag* real-time RT-PCR. The percentage of captured infectious virions (iVirion) or total virions captured (rVirion) were calculated independently with different denominators as follows: iVirion = [(100- flow-through infectivity) / (virus no-Ab infectivity)] x 100% and rVirion = [captured viral RNA copies / (captured viral RNA + flow-through viral RNA) x 100%.

### Mucosal IgG

Purified genital mucosal IgG from HIV-1+ women was prepared as described [16].

### HIV-1-Specific Binding Antibody Assay

Binding of mucosal antibodies to HIV-1 Env proteins were measured by a custom HIV-1 binding antibody multiplex assay as described [3, 4, 60–63]. Env proteins used included gp41, gp120 (clade B BaL), gp140 (consensus M ConS), V1/V2 loop (clade B CaseA V1/V2), and resurfaced core (RSC) proteins revealing the CD4bs (RSC3, and mutants RSC3Δ371, RSC3G367R, RSC3Δ371P363N abolishing CD4bs antibody binding [64]). RSC proteins were a kind gift from Dr. John Mascola. Total IgG and IgA Ab measurements for calculating specific activity were performed using Bio-Plex Pro human isotyping 7-plex panel (Bio-Rad) according to the manufacturer’s instructions.

### Data Analysis

Statistical analyses were performed in SAS 9.4 (SAS Institute, Cary NC) Differences in antibody-mediated virion internalization among isotypes and subclasses were determined by Sign test or by a linear mixed effects model [PROC MIXED]. Control for False Discovery Rate (FDR) was performed using the Benjamini and Hochberg method [65]. For all statistical comparisons, where applicable, results from separate assays using the same primary monocyte donor were averaged and treated as a single data point. Box plots and dot plots were graphed using GraphPad Prism (GraphPad Software Inc., San Diego CA).

## Results

### HIV-1 IgA Mediated Phagocytosis by Primary Monocytes via FcαRI

**Fig 1:**
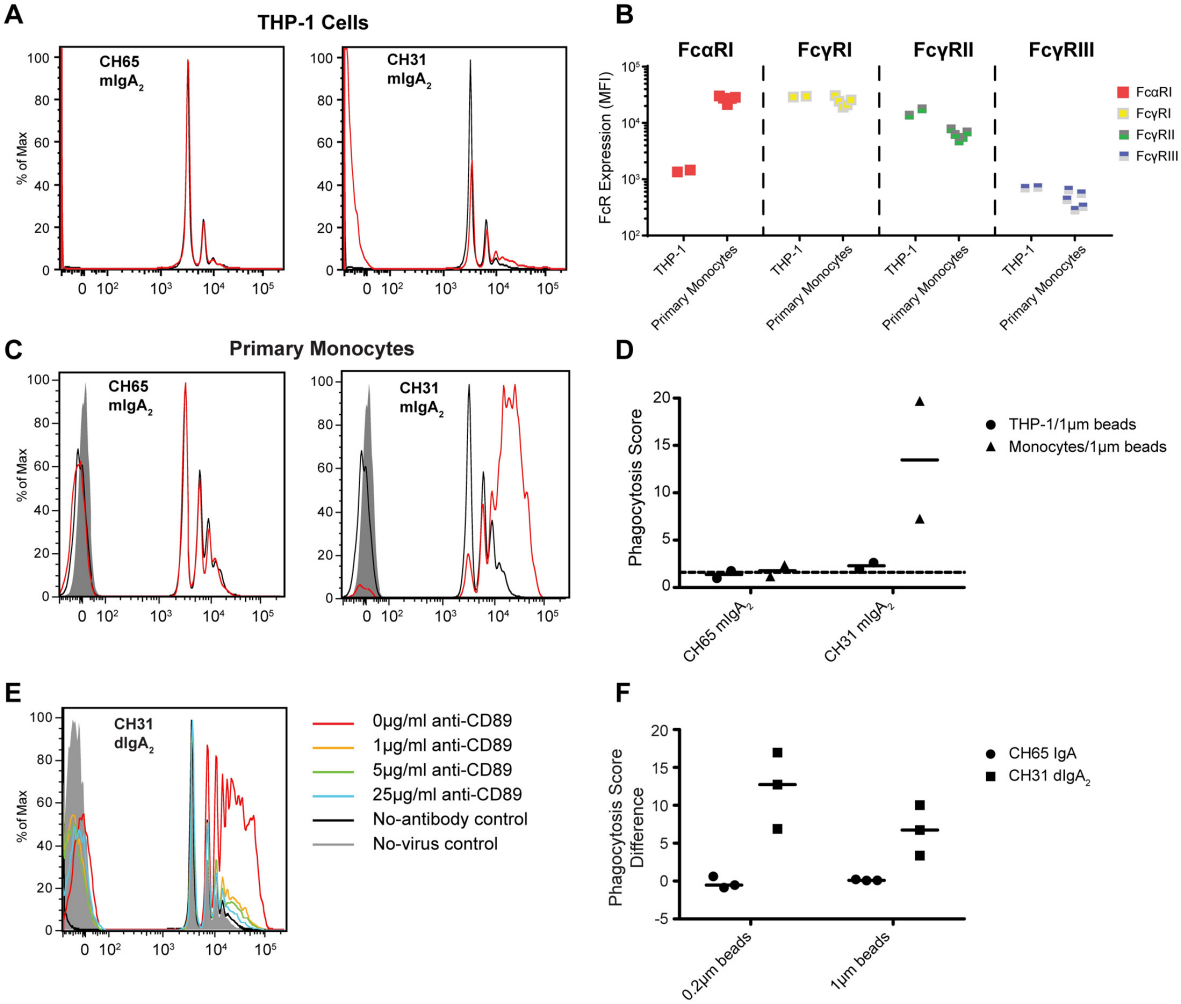
HIV-1 IgA mediates phagocytosis of beads and virions in primary monocytes through FcαRI (CD89). **A.** To investigate the ability of IgA to mediate phagocytosis in THP-1 cells, immune complexes were prepared *in vitro* by mixing IgA with ConSgp140-conjugated 1 μm fluorescent beads. Immune complexes were then added to THP-1 cells, and the uptake of IgA-ConSgp140 immune complexes was analysed by flow cytometry. Representative histograms of bead uptake by THP-1 cells for CD4bs bNAb CH31 mIgA2 and negative control anti-influenza mAb CH65 mIgA2 phagocytosis activity are shown. Red traces represent antibody-mediated internalization of beads, while the black trace represents background internalization of beads in the absence of antibody, and the grey solid area is the negative control without inclusion of beads. **B.** THP-1 cells and primary monocytes were phenotyped for expression of FcαRI, FcγRI, FcγRII, and FcγRIII by fluorescent antibody staining and flow cytometry. Compensated MFI values are reported (N = 2 independent experiments for THP-1 cells, N = 5 independent experiments for primary monocytes representing 5 different primary monocyte donors). **C-D.** Phagocytosis of IgA/ConSgp140 1μm bead immune complexes by primary monocytes is shown by a representative histogram of bead uptake for CD4bs bNAb CH31 mIgA2 and anti-influenza mAb CH65 mIgA2 phagocytosis activity (C). Bead phagocytosis was quantified using a phagocytosis score (see Methods) (D). The dashed line indicates background phagocytosis levels, measured by the mean + 3 SD of relevant negative controls. Results from 2 independent experiments are shown. **E-F.** To identify whether blocking of FcαRI reduces IgA-mediated phagocytosis, primary monocytes were incubated with various concentrations of anti-FcαRI (CD89) antibody for at least 1.5 hours at 4°C before addition to immune complexes made from 1 μm or 0.2 μm ConSgp140-conjugated fluorescent beads. Representative histograms of 1 μm bead uptake by monocytes in the presence of CH31 dIgA_2_ at 0, 1, 5, and 25 μg/ml anti-CD89 are shown (E). Anti-CD89-mediated blocking of antibody-mediated phagocytosis was quantified by taking the difference in phagocytosis score between experiments conducted in the presence of 5 μg/ml and 0 μg/ml anti-CD89 (F). Results from 3 independent experiments are shown.

Antibody-mediated phagocytosis of infected cells, and antibody-mediated internalization of virions exert important antiviral activity for a number of pathogens [25–35, 66]. For HIV-1, much is unknown about antibody-Fc mediated phagocytosis. Since HIV-1 infection occurs predominantly via the mucosal route, and since IgA plays an important role in mucosal immunity, we examined the ability of HIV-1 specific IgA to mediate phagocytosis of HIV-1 Env ConSgp140-conjugated beads. However, using the monocytic THP-1 cell line in which this phagocytosis assay was developed for HIV-1 [50], we observed very low levels of phagocytic activity, as shown by low levels of phagocytic activity mediated by a HIV-1 specific broadly neutralizing, CD4 binding site (CD4bs) antibody CH31 in a monomeric IgA2 (mIgA2) backbone (Fig 1A) [55].

Since antibody-dependent phagocytosis is dependent on engagement of Fc receptors, we hypothesized that THP-1 cells might express a different Fc receptor profile compared to primary monocytes, and that aberrant expression of the IgA Fc receptor, FcαRI (CD89), on THP-1 cells might have caused the lack of IgA-mediated phagocytic activity. Indeed, phenotyping the Fc receptor (FcR) expression of monocytic THP-1 cells as well as freshly isolated blood derived monocytes from HIV-1 negative healthy donors, we observed that monocytes and THP-1 exhibited a different FcR profile (Fig 1B). For the IgA receptor FcαRI (CD89), lower expression was found on THP-1 cells compared to primary monocytes (median MFI 1.40 x10^3^ and 2.8 x10^4^, respectively). THP-1 and primary monocytes expressed similar high levels of FcγRI (CD64) (median MFI 2.9 x10^4^ and 2.4 x10^4^ respectively). In contrast, THP-1 cells expressed 2.5-fold higher levels of FcγRII (CD32) than primary monocytes (median MFI 1.6 x10^4^ and 6.2 x10^3^, respectively). Both THP-1 cells and primary monocytes expressed very low levels of the IgG receptor CD16 (FcγRIII) (median MFI of 0.7 x10^3^ and 0.4 x10^3^ respectively), similar to previous reports for classical monocytes [67]. These results suggest that using infectious HIV-1 virions in primary phagocytes instead of HIV-1 envelope-conjugated beads in THP-1 may better mimic *in vivo* conditions and thus provide more precise information about antibody-mediated virion internalization.

As expected, IgA-mediated phagocytosis activity was much higher when primary monocytes were the phagocytes in the assay instead of THP-1 cells. The HIV-1 specific, CD4bs antibody CH31 [55] mIgA2 demonstrated higher HIV-1 envelope (ConSgp140CFI) conjugated bead uptake in primary monocytes (median phagocytosis score of 13.5) compared to THP-1 cells (median phagocytosis score of 2.3) (Fig 1C and 1D), likely due to the lower expression of FcαRI in THP-1 cells (Fig 1B). As expected, the non-HIV-1 antibody, CH65 mIgA2 [57], did not mediate phagocytosis of HIV-1 Env-coated beads (Fig 1C and 1D). Thus, a broadly neutralizing antibody with CD4bs specificity, CH31 [55], when in the IgA isotype, is capable of mediating phagocytosis.

In order to further verify that HIV-1 specific IgA-mediated phagocytosis is achieved via an FcαRI-dependent mechanism, we blocked FcαRI (CD89) by pre-incubating monocytes for at least 1.5 hr with anti-human CD89 prior to initiating phagocytosis. CD89 blocking abrogated HIV-1 specific IgA-mediated phagocytosis in monocytes. For ConSgp140CFI-conjugated 1 μm beads, a dose-dependent response was observed, with blocking of 78%, 74%, and 70% of phagocytosis by 25 μg/ml, 5 μg/ml, and 1 μg/ml of anti-CD89 mAb respectively (Fig 1E). When bead size was reduced to 0.2 μm, a size closer to that of HIV virions, 91.5% blocking of phagocytosis was observed with 5 μg/ml of anti-CD89 mAb (Fig 1F). Thus, HIV-1 IgA-mediated phagocytosis in primary monocytes is largely mediated by FcαRI.

### IgG1 Is More Potent Than IgA1 and IgA2 for Internalization of Infectious HIV-1 Virions

**Fig 2:**
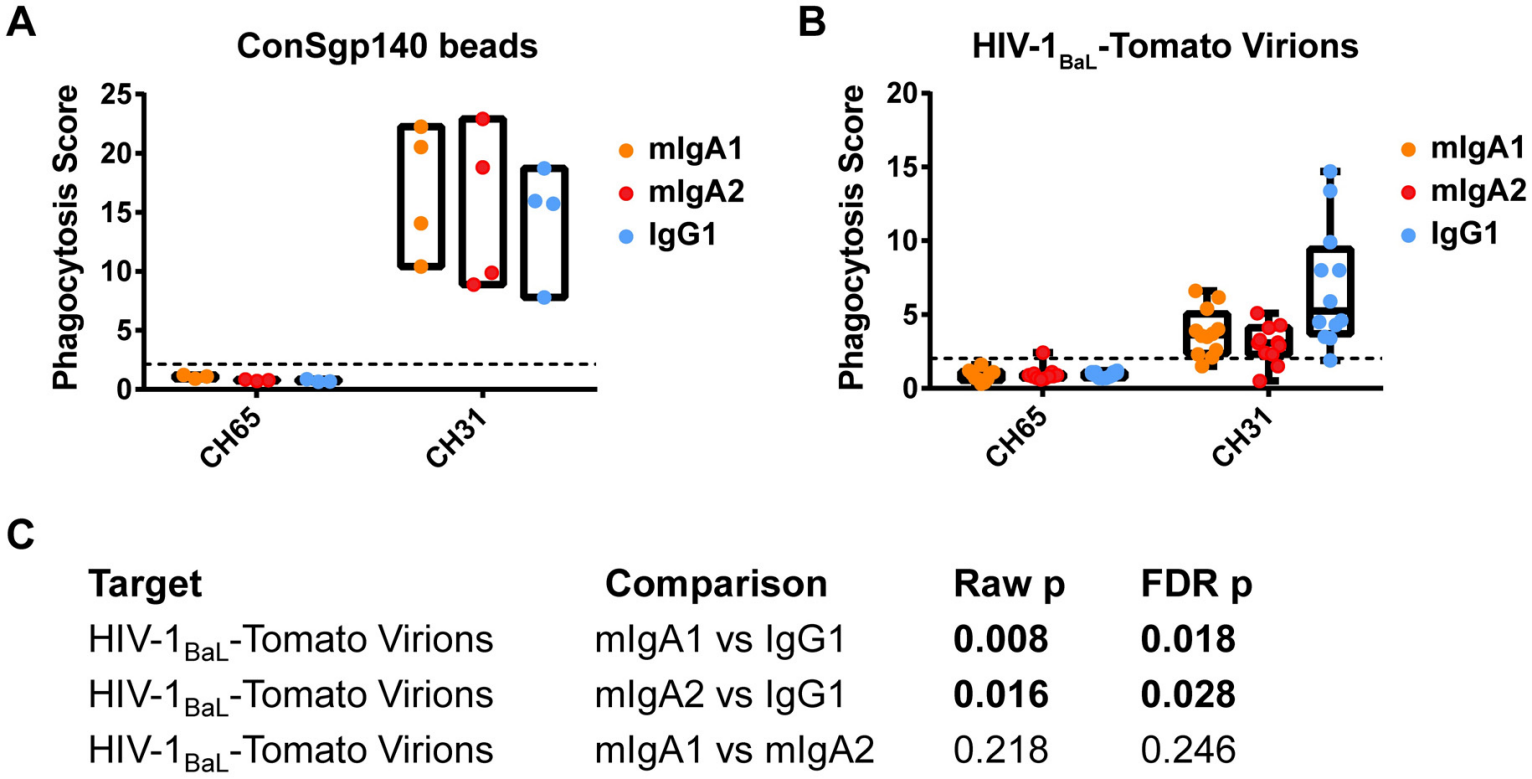
IgG1 mediates greater phagocytosis than mIgA1 and mIgA2 for virions but not for beads. **A-B.** To compare phagocytosis efficiencies of different immunoglobulin isotypes, mIgA1, mIgA2 and IgG1 was incubated with ConSgp140-conjugated 1 μm fluorescent beads (N = 4 independent experiments representing 2 different donors) (A) or with HIV-1_BaL_-Tomato virus (N = 10 independent experiments representing 8 different donors) (B), and the uptake of immune complexes by primary monocytes was analysed by flow cytometry. Box plots represent the range of phagocytosis scores, while box-and-whisker plots indicate 25^th^ and 75^th^ percentiles by box and minimum and maximum scores by whisker. Horizontal black dashed lines indicate limit of detection, as calculated using the mean + 3 SD of negative controls in the corresponding assays. **C.** The differences in phagocytosis score among immunoglobulin isotypes mIgA1, mIgA2, and IgG1 were compared pairwise using a Sign test.

Watkins *et al*. reported that HIV-1 dIgA1 provides better protection in SHIV mucosal challenge model compared to IgG1 and dIgA2 [46] and this protection was likely due to recognition of infectious virus particles. Subsequently, Sholukh *et al*. presented that IgG1+ dIgA2 conferred protection in a mucosal challenge model [47]. In order to determine the ability of different isotypes and subclasses of HIV-1 specific antibody to mediate phagocytosis, we expressed the CH31 CD4bs antibody [55] in an IgG1, mIgA1, or mIgA2 Fc expression construct [68] and examined their capacity for mediating phagocytosis of HIV Env-conjugated beads. All three forms of CH31 mAb mediated similar levels of phagocytosis of ConSgp140CFI-conjugated fluorescent beads in primary monocytes (Fig 2A).

Antibody-dependent internalization processes vary depending on the target’s properties [69–76]. Given the differences in size, rigidity, and antigen density between HIV-1 virions and HIV-1 Env-conjugated 1μm beads, we hypothesized that differences in phagocytic potency between IgG and IgA might only be apparent using virions. To date, the ability of HIV-1 specific antibodies to mediate internalization of infectious HIV-1 virions has not been characterized. Thus, to investigate this effector function, we developed a flow cytometric assay capable of demonstrating direct internalization of infectious HIV-1 virions. To allow visualization of internalized HIV-1 virions, they were labelled with the fluorescent proteins mcherry or Tomato [52]. Both THP-1 cells and primary monocytes were capable of internalizing HIV-1 virions in an antibody-specific manner (S1A and S1B Fig). Internalization was evident despite treatment of cells with 0.05% trypsin for 10 minutes after phagocytosis incubation to lyse surface-bound, uninternalized virus (S1C Fig).

All three forms of CH31 mAb mediated uptake of HIV-1 virions. In contrast to our previous results with Env-conjugated beads, when using infectious HIV-1 virions as the target antigen, IgG1 showed higher phagocytosis potency than IgA. This was shown by the 1.6-fold higher phagocytosis score for CH31 IgG1 (median phagocytosis score 5.3) than CH31 IgA1 (median phagocytosis score 3.6) (FDR_p = 0.018), and 2.1-fold higher phagocytosis score for CH31 IgG1 than CH31 IgA2 (median phagocytosis score 3.0) (FDR_p = 0.028) (Fig 2B and 2C). CH31 IgA1 showed a non-significant trend toward higher phagocytosis potency than IgA2 (FDR_p = 0.246). Thus, IgG1 is more potent than IgA in mediating virion internalization.

### IgG3 Is More Potent Than IgG1 for HIV-1 Bead and Virion Internalization

**Fig 3:**
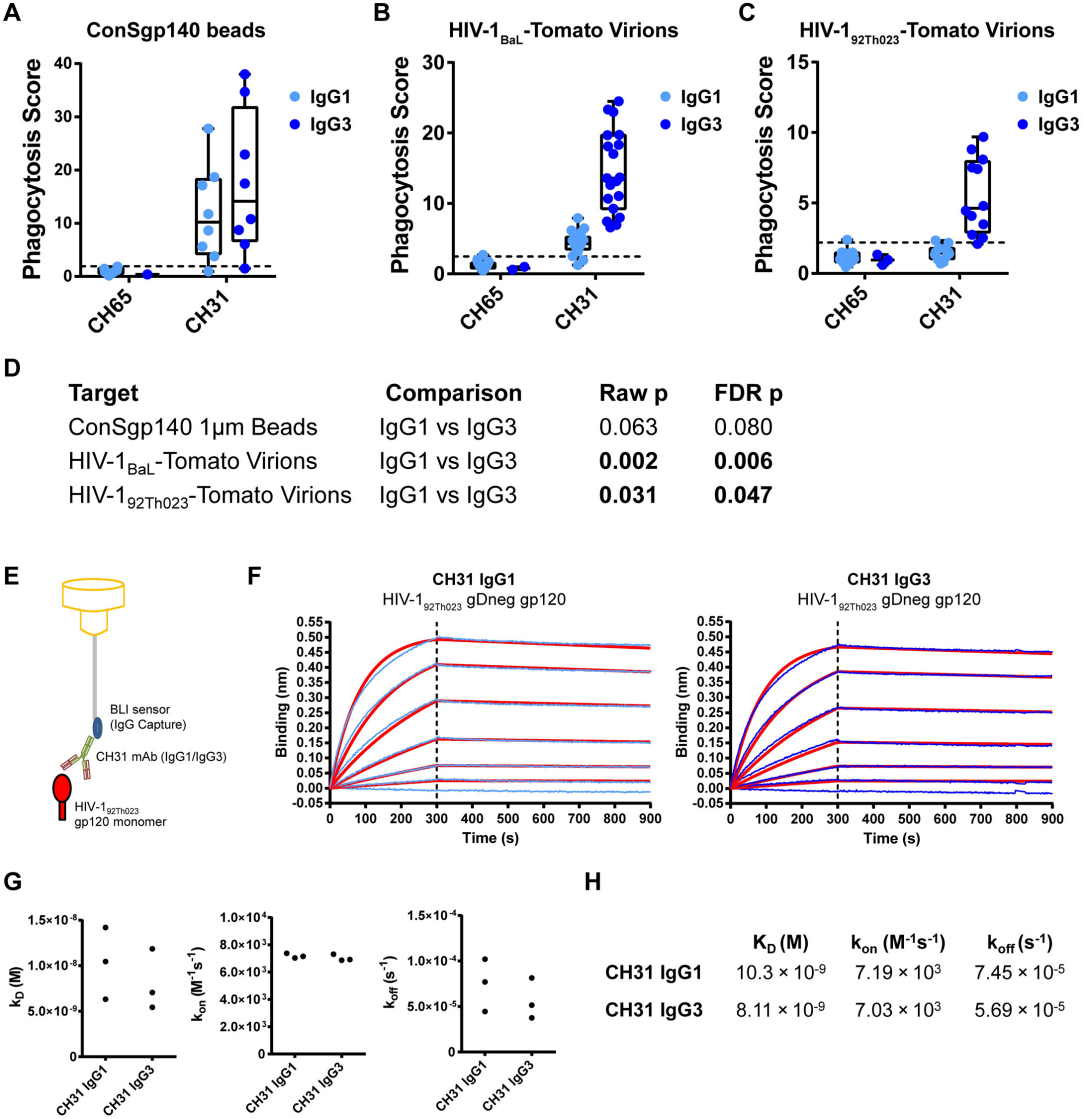
IgG3 shows greater HIV-1 virion internalization than IgG1, independent of Env protein binding. **A-D.** Wild type CH31 IgG3 and IgG1 were tested for internalization of ConSgp140-conjugated 1 μm fluorescent beads (N = 8 independent experiments representing 6 different donors) (A), HIV-1_BaL_-Tomato virions (N = 19 independent experiments representing 11 different donors) (B) and HIV-1_92TH023_-Tomato virions (N = 12 independent experiments representing 6 different donors) (C) in human primary monocytes. Anti-influenza mAb CH65 in each subclass backbone were also tested as negative controls. Box-and-whisker plots indicate 25^th^ and 75^th^ percentiles by box, and minimum and maximum scores by whisker. Horizontal black dashed line indicates limit of detection, as calculated using the mean + 3 SD of negative controls in the corresponding assays. The differences in phagocytosis score were compared between IgG1 and IgG3 using a Sign test (D). **E-H.** To examine if differences in phagocytosis were due to different binding to HIV-1 Env, antibody binding to HIV-1 Env protein was tested using biolayer interferometry. Antibodies (CH31 and CH65 IgG1 and IgG3) were loaded on a Human IgG Capture sensor, and binding to HIV-1_92Th023_ gDneg gp120 monomer protein in solution was tested (E). Specific binding curves of gp120 binding to CH31 IgG1 and IgG3 (light blue and dark blue lines respectively) are shown along with 1:1 Langmuir model fitted curves (red lines) (F). Dissociation constant (K_D_), association rate (k_on_), and dissociation rate (k_off_) are shown for 3 independent experiments (G), and their respective median values are also shown (H).

To determine the impact of IgG subclass on capacity to mediate antibody-dependent virion internalization, the broadly neutralizing CD4bs antibody CH31 [55] was generated in different Fc backbones as recombinant IgG1 (wild-type SEK) or IgG3 (wild-type), and the resulting antibodies were compared for potency in internalization of ConSgp140-conjugated 1μm beads (Fig 3A), HIV-1_BaL_-Tomato virions (Fig 3B), and HIV-1_92TH023_-Tomato virions (Fig 3C). CH31 IgG3 showed a non-significant trend (FDR_p = 0.080) toward higher phagocytosis of ConSgp140-conjugated 1 μm beads (median phagocytosis score 14.1) compared CH31 IgG1 (median 10.2) (Fig 3A and 3D). CH31 IgG3 showed significantly higher phagocytosis of HIV-1_BaL_-Tomato virions (median phagocytosis score 13.6), with 3.8-fold higher phagocytosis score than CH31 IgG1 (median 4.3) (FDR_p = 0.006) (Fig 3B and 3D). The same pattern was observed for internalization of HIV-1_92Th023_-Tomato virions, where CH31 IgG3 showed significantly higher phagocytosis of HIV-1_92Th023_-Tomato virions (median phagocytosis score 4.6), with 7.8-fold higher phagocytosis score than CH31 IgG1 (median 1.5) (FDR_p = 0.047) (Fig 3C and 3D). Thus, IgG3 is more potent than IgG1 for HIV-1 bead and virion internalization (Fig 3D).

### Phagocytic Activity by Antibody Isotypes/Subclasses Is a Distinct Immune Measurement from Binding to Soluble Envelope Proteins

To determine whether the variance in the virion internalization by different antibody isotypes/subclasses was caused by the ability to bind HIV-1 envelope, CH31 IgG1 and IgG3 were tested for their binding to HIV-1_92Th023_ gp120 protein by biolayer interferometry (Fig 3E and 3F). In contrast to the results observed for virion internalization, we observed that the K_D_ values for CH31 IgG3 (mean K_D_ = 8.1 x 10^−9^ M) and CH31 IgG1 (mean K_D_ = 10.3 x 10^−9^ M) were similar (less than two-fold difference) (Fig 3G and 3H). Thus, CH31 IgG3 did not mediate increased binding to HIV-1 Env compared with CH31 mIgG1. Similar results were obtained with and without subtraction of negative control CH65 IgG3 and IgG1 non-specific binding respectively, since non-specific binding levels of CH65 to HIV-1_92Th023_ gp120 antigen were low. These results indicate that the HIV-1 virion internalization assay distinguishes differences between antibody isotypes and subclasses in mediating phagocytosis distinct from HIV-1 envelope binding.

### Phagocytic Activity of Antibody Isotypes/Subclasses Confirmed by ImageStream Cytometry

**Fig 4:**
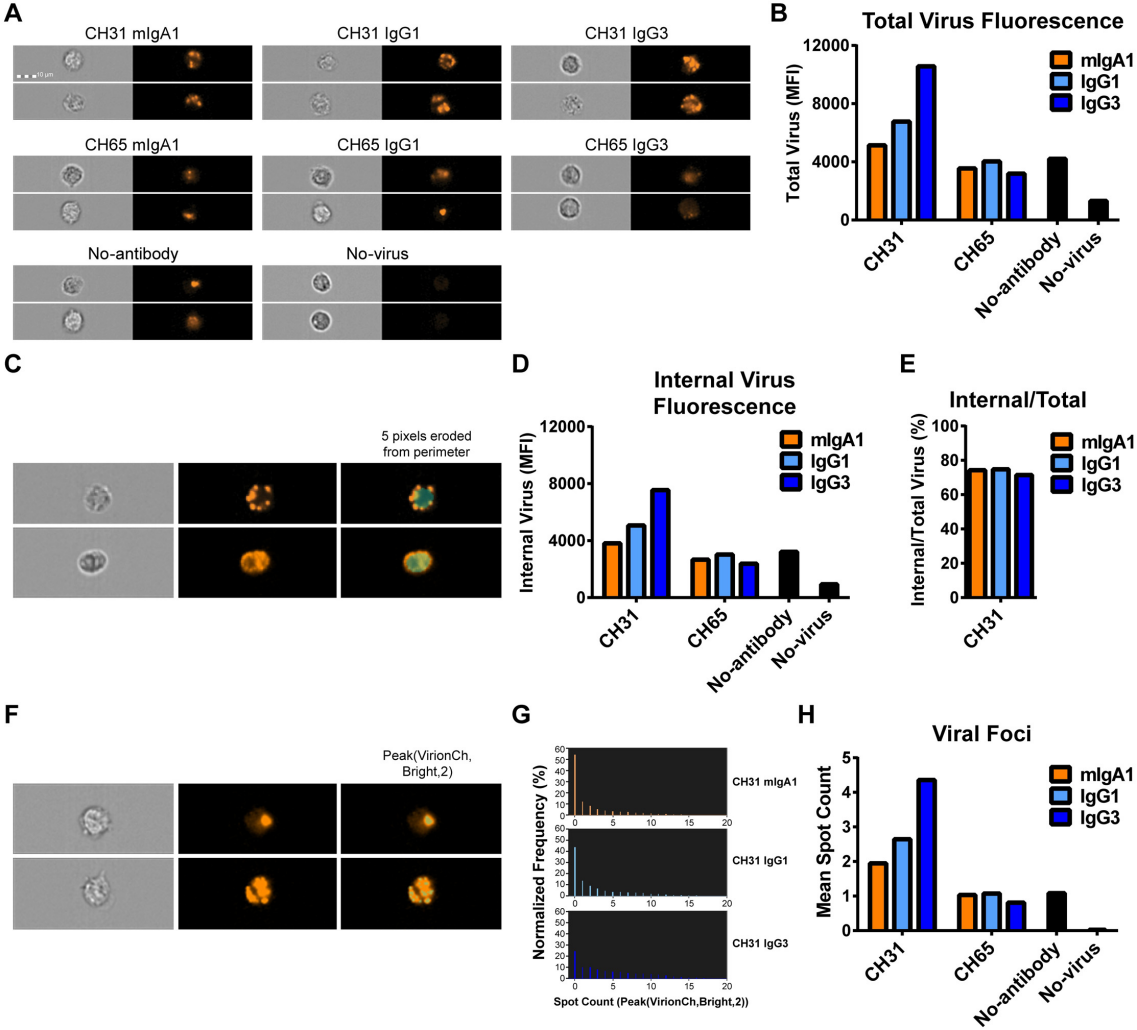
ImageStream imaging of IgG and IgA-mediated virion internalization shows distinct internalized virus puncta. **A.** Fluorescent infectious HIV-1_BaL_-Tomato virions were spinoculated and incubated with freshly isolated monocytes and antibodies for antibody-mediated virion internalization to occur. Virion internalization was visualized with ImageStream^X^ Mark II (EMD Millipore), collecting more than 10,000 images per setup. Representative images are shown for the CH31 CD4bs bNAb antibody engineered in IgG3, IgG1, and mIgA1 backbones, control anti-influenza CH65 antibodies, and two control conditions without antibody and without virus/antibody respectively. **B.** Total virus fluorescence was quantified for each antibody. Virus fluorescence was quantified using the mean fluorescence intensity of all single, focused cell images for each antibody (~5,000 images each). **C.** To exclude surface-bound virions, a mask was applied to demarcate the internal portion of the cell, as defined by the erosion of 5 pixels into the bright-field perimeter of the cell. Two representative cells are shown, the upper row showing a cell with mostly excluded surface-localized virus, and the lower row showing a cell with both surface and deep internalized virus (left, bright-field; middle, virus fluorescence; right, virus fluorescence with blue mask demarcating internal portion of the cell). **D.** Internal virus fluorescence was quantified for each antibody condition. Virus fluorescence was quantified using the mean fluorescence intensity of all single, focused cell images for each antibody (~5,000 images each). **E.** The percentage of fluorescence intensity comparing the 5-pixel-eroded image to the original image is shown for each CH31 antibody form. **F.** To count viral foci, a mask determined by the ImageStream IDEAS Spot Wizard algorithm was applied, representing the areas with peak brightness defined by a spot-to-background ratio of 2.0. Spots within this mask were counted. Two representative cells are shown, the upper row showing a cell with 1 virus foci, and the bottom row showing a cell with 14 virus foci (left, bright-field; middle, virus fluorescence; right, virus fluorescence with blue mask demarcating the applied mask for peak brightness). **G.** The distribution of spot counts is shown for the cells in each CH31 antibody condition. **H.** The mean number of viral foci is shown for each condition.

To confirm IgG1, IgG3 and IgA-mediated internalization of infectious HIV-1 virions by primary monocytes, we examined virion internalization by ImageStream cytometry, a technique combining flow cytometry and fluorescence microscopy. The phagocytosis assay was set up similar to previous experiments, except that fixed cells were visualized on an ImageStream cytometer to obtain more than 10,000 cell images per condition. We observed internalization of virus within monocytes, seen as distinct intracellular fluorescent puncta (Fig 4A). Consistent with the results obtained by flow cytometry, CH31 IgG3 showed greater virion internalization than CH31 IgG1 and CH31 mIgA1, with higher mean intensity in the virus fluorescence channel (10.5 x 10^3^, 6.8 x 10^3^, and 5.1 x 10^3^ MFI, respectively) (Fig 4B). Values for all CH31 antibodies remained above all negative controls.

To examine whether these differences were due to surface binding of virions, we calculated the intensity of fluorescence within only the internal portion of the cell, as defined by the area remaining after eroding 5 pixels into the perimeter of the bright-field mask (Fig 4C). The erosion of pixels from cell images to identify the internal area of the cell is a part of the standard data analysis software (“Internalization Wizard”) for the ImageStream instrument [77–79]. Since the acquired cells are gated on image focus, analyzed cells fall within a particular depth range at which the acquired image represents a cross-section of the cell with consistent membrane thickness [80]. We additionally confirmed this technique by showing that surface-binding fluorescent probes have less fluorescence excluded after erosion compared to internal probes (S2A Fig). After the 5-pixel erosion, some fluorescence was lost in all conditions, since intensity is the sum of all fluorescence observed within the measured area, which decreased. However, the pattern observed between antibody isotypes and subclasses remained unchanged, with CH31 IgG3 having the highest mean virus fluorescence intensity (7.5 x 10^3^ MFI), followed by CH31 IgG1 (5.1 x 10^3^ MFI), then CH31 mIgA1 (3.8 x 10^3^ MFI), and these remained higher than the negative controls (Fig 4D). Furthermore, the percentage of fluorescence lost as the image was eroded was consistent across antibody isotypes/subclasses, indicating that average depth of internalization was similar (Fig 4E, S2B Fig). Thus, ImageStream analysis confirms the antibody-mediated internalization of virus particles.

As an additional quantitative measure of virion phagocytosis, the number of viral foci per cell was counted using the Spot Wizard. To define the stringency criteria for the mask used for spot counting, the ImageStream IDEAS Spot Wizard algorithm was trained using two groups of at least 25 focused, single cells that were manually classified—one with low spot numbers and one with high spot numbers. Images of varying spot sizes, shapes, intensities, and backgrounds were used in each category. This yielded a mask that comprised the Peak areas (defined by a spot-to-background ratio of 2.0) of the Bright mask in the virion fluorescence channel (Fig 4F). This yielded different distributions of spots for CH31 IgG3, CH31 IgG1, and CH31 mIgA1 (Fig 4G). CH31 IgG3 had the highest mean spot count of 4.35, followed by CH31 IgG1 with a mean spot count of 2.64, followed by CH31 mIgA1 with a mean spot count of 1.95 (Fig 4H). All CH31 antibody conditions showed higher spot counts than the negative controls. Thus, antibody isotypes/subclasses display differences in number of viral foci per cell in addition to differences in total virion internalization.

### IgG3 Mediates Enhanced Phagocytosis Across Multiple HIV-1 Epitopes

**Fig 5:**
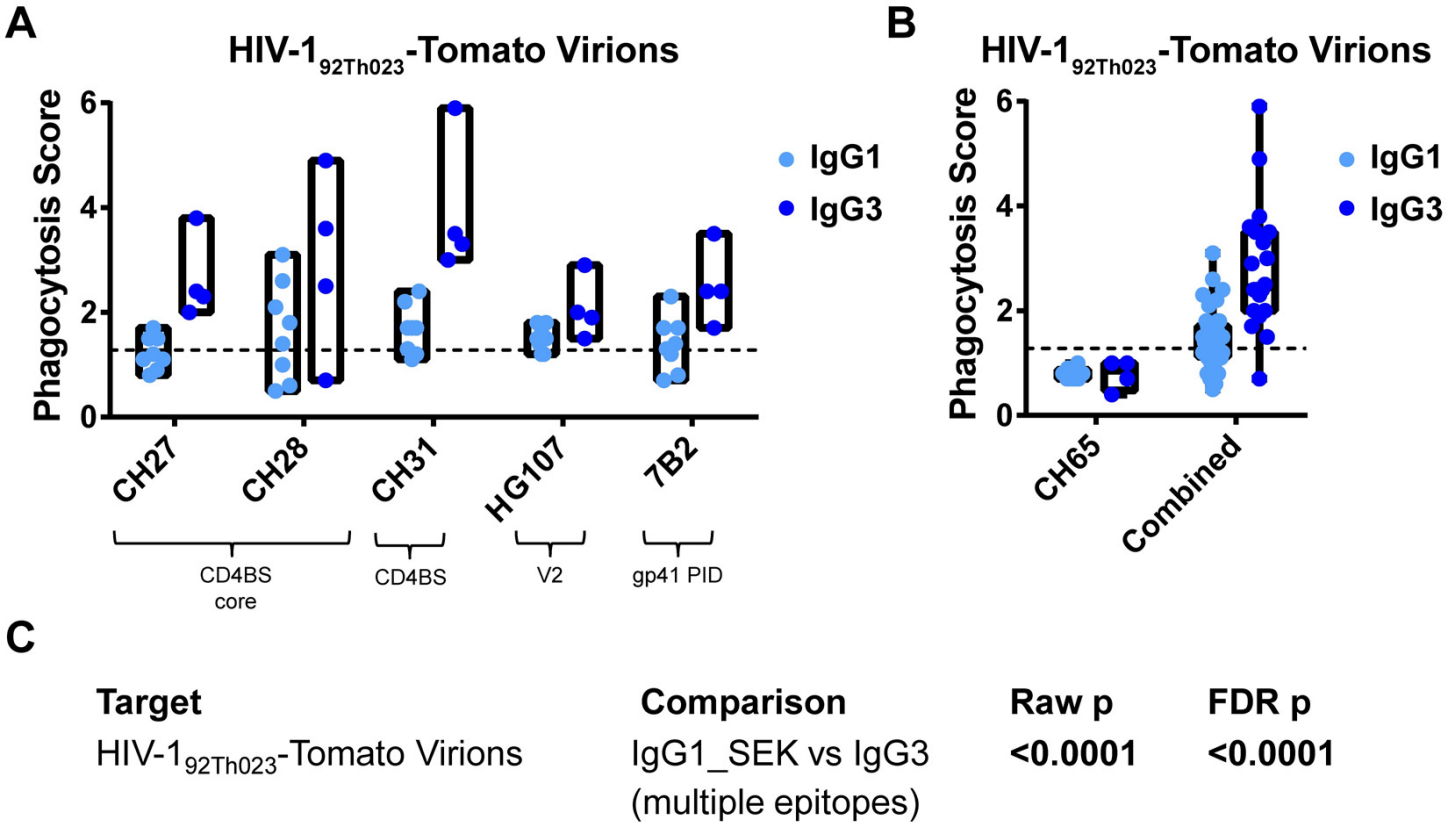
IgG3 has enhanced phagocytosis potency across multiple HIV-1 epitopes. **A.** Epitope-matched IgG3 and IgG1 mAbs were tested for HIV-1_92TH023_-Tomato virion phagocytosis in human primary monocytes. Phagocytosis-positive antibodies are shown (N = 4 independent experiments). Box plots represent the range of phagocytosis scores. Horizontal black dashed line indicates limit of detection, as calculated using the mean + 3 SD of negative controls in the corresponding assays. **B.** Data from antibody paratopes positive for phagocytosis (CH27, CH28, HG107, 7B2, CH31) were aggregated by subclass. Box-and-whisker plots indicate 25^th^ and 75^th^ percentiles by box and minimum and maximum scores by whisker. **C.** The differences in phagocytosis score were compared between IgG1_SEK and IgG3 using a linear mixed effects model.

In order to identify whether the observed phenomenon of enhanced IgG3 phagocytosis potency was applicable to epitopes other than the CD4 binding site epitope, we generated epitope-matched IgG1 and IgG3 antibodies for multiple HIV-1 epitope specificities, including the gp41 principal immunodominant domain (gp41 PID), a gp41 conformational epitope, the V1/V2 loop, the CD4 binding site core, and the C1 conformational epitope. Additionally, two IgG1 antibody forms were produced for each epitope—a wild type (SEK) and an ADCC-optimized (AAA/4A) form [58, 59]. We tested these antibodies for phagocytosis of HIV-1_92Th023_-Tomato virions. No differences were found between the two IgG1 forms, so results from IgG1_SEK and IgG1_AAA/4A were aggregated. All antibodies that were positive for phagocytosis in the IgG1 backbone showed higher phagocytosis scores in the IgG3 backbone (Fig 5A and 5B). Specifically, for broadly neutralizing antibodies CH27, CH28 (gp120 CD4-binding site core-specific) [56], non-broadly neutralizing antibodies HG107 (V1/V2-specific, isolated from an RV144 vaccinee) [14] and 7B2 (gp41 PID-specific) [7], IgG3 showed enhanced internalization of infectious HIV-1_92TH023_-Tomato virions (median phagocytosis scores 2.0 to 3.1) compared to their IgG1 counterparts (phagocytosis score 1.2 to 1.7), with an overall 3.2-fold higher phagocytosis score for IgG3 compared to IgG1. For mAbs targeting epitopes not present on virions, such as the C1-conformational epitope and the gp41-conformational epitope, neither IgG3 nor IgG1 internalized virions. Aggregating the paratopes that were positive for virion internalization (CH27, CH28, HG107, 7B2, CH31) allowed statistical comparison of IgG3 and IgG1_SEK subclasses for HIV-1_92TH023_-Tomato virion internalization using a linear mixed effects model, which showed that IgG3 has significantly higher phagocytosis potency than IgG1_SEK across multiple epitopes (p<0.0001) (Fig 5C).

### HIV-1 + Mucosal IgG Mediates Virion Internalization

**Fig 6:**
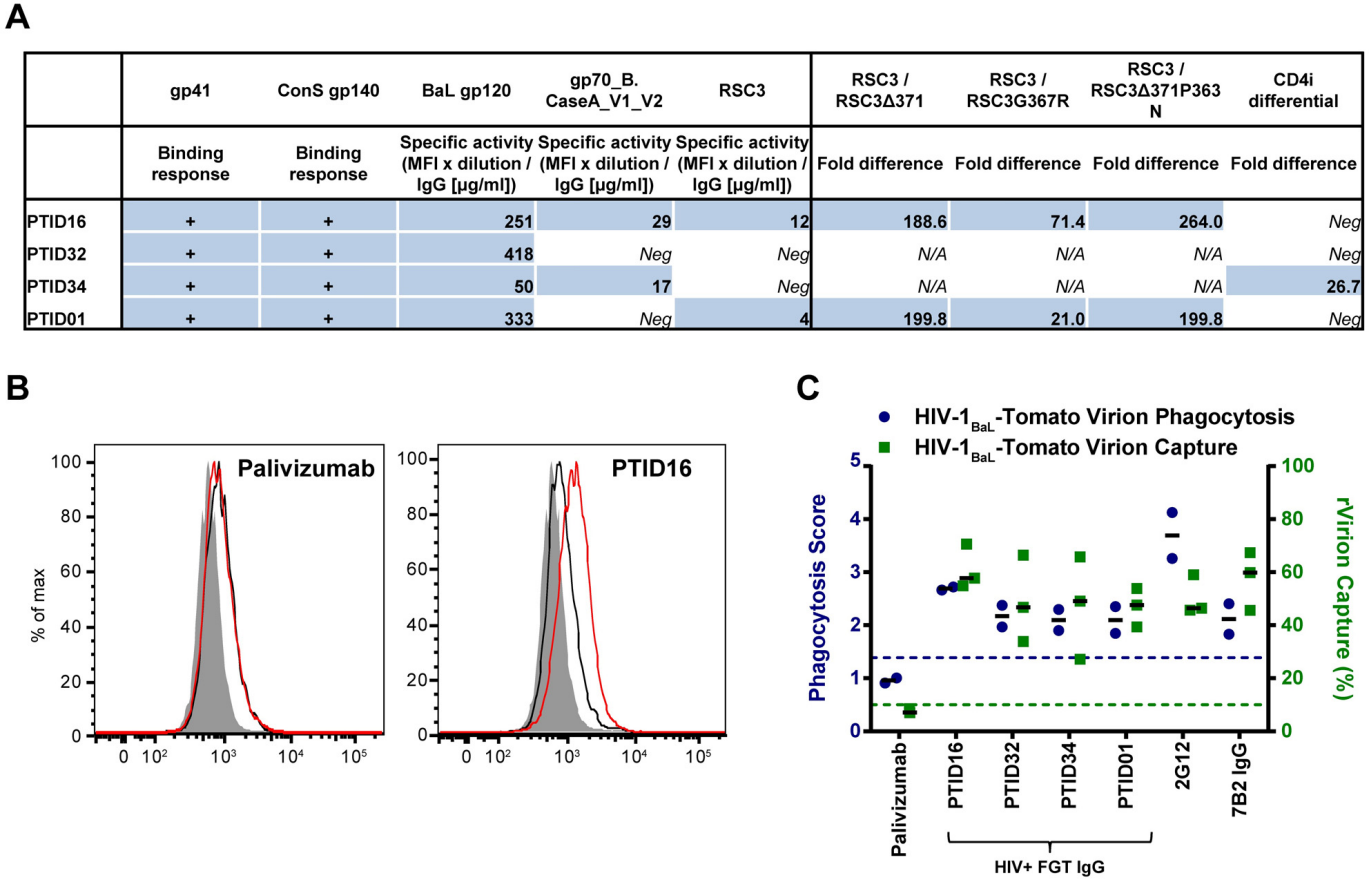
Mucosal HIV-1 specific polyclonal IgG from vaginal wecks from HIV-1+ women can capture virions and mediate internalization of infectious HIV-1_BaL_. **A.** HIV-1 envelope binding profile is shown for purified IgG of 4 HIV-1+ women (PTID16, PTID32, PTID34, PTID01) positive for infectious and non-infectious virus capture. Binding responses to gp41 and ConSgp140 reached saturation. Specific activity for BaL gp120, gp70_B.CaseA_V1_V2, and RSC3 is shown, and samples with FI-background<100 were classified negative. Classification of VRC01 like CD4bs binding antibodies is indicated by the ratio of binding MFI of the CD4bs-exposed RSC3 to CD4bs mutants RSC3Δ371, RSC3G367R, and RSC3Δ371P363N, and classification of CD4i antibodies is indicated by CD4i differential, the ratio of binding MFI of HxB2 core to HxB2 core I420R. **B.** The ability of the mucosal HIV-1+ purified IgG samples to mediate uptake of HIV-1_BaL_-Tomato by THP-1 cells was analysed by flow cytometry. A representative flow cytometry diagram of virion internalization mediated by mucosal IgG isolated from a vaginal weck from a HIV-1+ woman (PTID16) is shown alongside a representative diagram for a negative control (RSV-specific Palivizumab). **C.** The ability of mucosal IgG in chronically infected women to mediate virus capture and virion internalization was quantified. Blue circles represent phagocytosis scores for HIV-1_BaL_-Tomato virions in THP-1 cells (N = 2 independent experiments), while green squares represent virus capture percentages as measured by RT-qPCR (N = 3 independent experiments).

To extend these findings from monoclonal antibodies to human clinical samples relevant for mucosal protection, we next tested whether HIV-1 specific mucosal IgG from the female genital tract could mediate virion internalization. We previously reported the capacity of mucosal IgG from vaginal wecks to bind infectious virions (i.e. virus capture) [16] and here we selected clinical samples with evidence of virion recognition to test for internalization by phagocytes. Thus, we isolated IgG from vaginal wecks from 14 HIV-1+ women to test for infectious virion capture and binding to different parts of the HIV-1 envelope, including gp41, gp120, gp140, the V1/V2 loop, and the CD4 binding site. Mucosal IgA was not tested, due to low yields of HIV-1 specific IgA in these samples, confirming previous reports [4, 81, 82]. 4/14 samples (total IgG concentration range: 50.2 μg/ml—129.2 μg/ml) showed both infectious and non-infectious virus capture. All four samples had high levels of gp41, gp120 and gp140-binding antibodies (Fig 6A). Two of the four participants (PTID16 and PTID34) had V1/V2 loop binding. Two (PTID16 and PTID01) showed CD4bs binding specificity, as represented by differential binding to resurfaced core protein (RSC3) compared to mutants that decrease VRC01-like CD4bs binding specificity (RSC3Δ371, RSC3G367R, RSC3Δ371P363N) [64]. Purified IgG from these four participants captured 47–61% of total HIV-1_BaL_ virus particles. Purified IgG from these four participants were then evaluated for their ability to mediate virion internalization. Indeed, HIV-1+ mucosal IgG mediated HIV-1_BaL_ virion internalization in monocytes, with median phagocytosis scores of 2.1 to 2.7 (Fig 6B and 6C). Thus, human mucosal IgG from HIV-1+ individuals are capable of mediating both virion capture and IgG-mediated internalization of infectious HIV-1, demonstrating proof of concept that easily elicited antibodies can mediate these potential antiviral effector functions at the portal of HIV-1 entry.

## Discussion

Substantial evidence suggests a role of Fc-receptor mediated antibody functions in protection against HIV-1 infection [3–12]. Here, we evaluated Fc-mediated HIV-1 virion internalization by primary monocytes. We demonstrated that HIV-1 envelope specific IgG and IgA can complex with infectious HIV-1 virions leading to phagocytosis by primary monocytes. The potency of virion internalization was modulated by antibody Fc, where IgG3 was the most potent, followed by IgG1, then IgA. Furthermore, we found that HIV-1 infection elicits mucosal IgG that can recognize infectious virions, providing proof of concept that these types of antibodies easily elicited by vaccination can provide partial blocking HIV-1 acquisition at mucosal surfaces. Further studies are needed to evaluate the potential protective and/or detrimental roles of this Fc-mediated antibody function at mucosal sites.

Given the importance of antibody isotype/subclass profile in HIV-1 vaccine efficacy [4–6, 44] and protection in mucosal challenge models [46, 47], we sought to determine differences in effector function among isotypes/subclasses. Here, we identified substantial differences in IgA, IgG1, and IgG3 in mediating virion internalization. Using both flow cytometry and ImageStream cytometry, we demonstrate enhanced virion internalization by IgG3 compared to IgG1, and reduced virion internalization by IgA1 and IgA2 compared to IgG1. A previous report of improved protective functions for IgA1 over IgA2 were determined with antibodies specific for the V3 region of the HIV-1 envelope glycoprotein and did not include examination of antibody-mediated phagocytosis [46]. Later work by this same group also demonstrated protective capacity for dIgA2 when combined with IgG1 [47]. Our work differs from these prior studies in that we examined a different epitope specificity (i.e. CD4bs vs. V3), monomeric vs dimeric forms of IgA, and a different type of antiviral function (phagocytosis vs. virion capture and transcytosis). Further work is needed to understand if the structure of the IgA (i.e. the longer hinge region of IgA1 over IgA2) is beneficial for some, but not all, epitope specificities and antiviral functions, depending on how occluded or exposed their target site is on the presenting pathogen.

We further generated additional monoclonal antibodies to test a broader panel of epitope specificities to determine whether these effects observed are specific to a particular epitope. IgG3 was more potent than IgG1 for all epitope specificities in mediating virion internalization, indicating that the observed effects were not epitope-dependent. Our identification of enhanced IgG3 mediated phagocytic function supports findings in both virus controllers [12] and in RV144 vaccinees [4, 5] that this antibody response is associated with a more functional antibody profile. However, unlike Chung et al. who concluded that IgG3 may only be a surrogate of a functional IgG1 response [6], our data present an additional potential role for direct IgG3 effector function via enhanced antibody-mediated phagocytosis activity, and raise the hypothesis that the correlation of Env IgG3 with reduced HIV-1 risk may be in part due to this improved Fc mediated antiviral function.

We found that the differences in virion internalization activity among antibody isotypes/subclasses were not associated with HIV-1 Env antigen binding, indicating that the observed differences in virion internalization were not simply due to differences in affinity for the target. The mechanisms behind the differences among antibody subclasses/isotypes to mediate virion internalization remain to be determined. Potential mechanisms can be divided into effects from paratope binding and effects from Fc receptor engagement by the antibody Fc region. Regarding paratope binding, antibody isotypes/subclasses differ in hinge length, flexibility, and the angle of their Fab arms, and these differences may result in avidity effects that lead to increased virion engagement and internalization, especially in light of the relatively few number of Env spikes on the HIV-1 virion surface. For example, IgG3 has more potent neutralization activity than IgG1 and IgG2, attributed to the longer and more flexible hinge region [83]. Other examples include studies showing that dimeric IgA1 better mediates virus capture compared to IgG1 and dimeric IgA2 [46], although IgG1 and monomeric IgA are equally capable of mediating virion aggregation [84]. Regarding differences in Fc receptor engagement, IgG and IgA engage different Fc receptors, but it is not known how the different signals contribute to internalization. Comparing IgG1 and IgG3, it is known that IgG3 shows stronger binding to several Fc receptors [48], which may have resulted in the observed enhancement in phagocytic activity. In understanding the contributions of each Fc receptor, it is notable that we observed no difference in virion internalization was observed between CH31 IgG1_SEK and CH31 IgG1_AAA antibodies (Fig 5A and 5B). Given that the AAA mutations are designed for optimal binding to FcγRIII [58, 59], it is unlikely that differences in phagocytic potency are contributed by binding to FcγRIII. This is consistent with the known low expression of FcγRIII on classical monocytes [67], as well as previous studies which showed that HIV-1 internalization by IgG is primarily mediated by the interaction of FcγRI and FcγRII, and not FcγRIII in primary monocytes and THP-1 cells [39, 50]. Given that the AAA mutation enhances ADCC, the finding that the AAA mutation does not enhance phagocytosis provides an example demonstrating that interactions which enhance one Fc-mediated function do not necessarily also enhance other Fc-mediated functions. Further studies of the relationship between FcR signalling and antibody-dependent internalization are needed to understand the differences in effector functions between antibody isotypes and subclasses.

Since monocytes/macrophages and neutrophils are the predominant Fc receptor bearing cells in the cervico-vaginal mucosa [37](personal communication, Drs. R Astronomo and MJ McElrath), we focused our evaluation of the antiviral potential of HIV-1 specific antibodies with primary monocytes. Future studies are needed to test mucosal vaginal macrophages and neutrophils that express FcαRI and FcγRI, II, and III to further understand the potential for engaging mucosal effector cells by vaccine-elicited antibody responses. IgA is the dominant antibody isotype in the gut mucosa, and we demonstrate that HIV-1 specific IgA mAbs could mediate infectious virion internalization by primary monocytes. Moreover, mucosal IgG purified from vaginal wecks of HIV-1 infected women mediated phagocytosis of virions. These data provide proof of concept that certain specificities of HIV-1 antibodies if elicited by vaccination could mediate phagocytosis of virus particles in the mucosa, representing an additional potential antiviral function.

Virion internalization was mediated by mucosal IgG from HIV-1 infected women that comprised a range of antibody epitope specificities, including V1/V2, gp41, and the CD4 binding site. Interestingly, the participant with the highest virion internalization score (PTID16) also had the highest CD4bs binding score among all 14 participants evaluated, suggesting that the CD4 binding site may be an important target for virion internalization at the mucosal surface. A larger study is required to examine the relationship between antibody epitope specificity and virion internalization.

Infected cells and virion particles are two biological targets of antibody-mediated internalization, but the role of each of these processes in HIV-1 infection is unclear. Advances are needed in characterizing each of these to inform strategies of HIV-1 prevention and cure. One potential method of separating these internalization processes is to utilize the difference in target size, which has been associated with different mechanisms of internalization, where smaller particles are taken up by endocytosis dependent on clathrin, Cbl activation, ubiquitinylation, and proteasome function, while larger particles are taken up by phagocytosis dependent on actin as well as activation of Src, Syk, and PI3K [69–71, 73, 74]. We found a similar dichotomy between smaller and larger particles, where smaller particles including 0.2 μm beads, 40 nm beads, and HIV-1 virions (~0.1 μm) had higher phagocytosis scores in primary monocytes compared to THP-1 cells, while larger particles (1 μm beads) had higher phagocytosis scores in THP-1 cells compared to primary monocytes (S3 Fig). These differing phenotypes may point toward different internalization mechanisms for viruses compared with beads, raising the hypothesis that the virus-based and bead-based assays represent phagocytosis of different kinds of targets *in vivo*. Given that when beads were used as internalization targets instead of virions, we observed no differences between IgG1 and IgA and a non-significant trend toward higher IgG3 than IgG1 phagocytosis potency (Figs 2A, 3A and 3D), antibody isotypes and subclasses may have differing effects in mediating cell and virion phagocytosis. Further work comparing the mechanisms of bead and virion uptake is needed to better understand the HIV-1 correlates of risk that have been identified in non-human primate models using the 1 μm bead-based phagocytosis assay [8, 10] to determine whether this represents phagocytosis of infected cells or virus particles.

We identified significant differences among the antibody isotypes/subclasses for capacity to mediate virion internalization, with Env IgG3 of multiple specificities having the highest potency, followed by IgG1, then IgA1 and IgA2. Moreover, HIV-1 specific IgG and IgA can mediate phagocytosis of infectious HIV-1 virions and IgG Fc mediated antiviral activity can be active at the vaginal mucosa. These data provide fundamental insights on Fc mediated antibody effector functions that may be important for protection against HIV-1 acquisition and for HIV-1 cure strategies.

## Supporting Information

S1 Fig HIV-1 IgG mediates internalization of HIV-1 virus. **A-B.** Internalization of infectious HIV virus was tested by incubating PG9 IgG/HIV-1_CM235_-mcherry immune complexes with THP-1 cells (A) or by incubating CH31 IgG/HIV-1_BaL_-Tomato immune complexes with human primary monocytes (B). Representative flow cytometry histograms of independent experiments (N = 2 and N = 8 respectively) are shown. Red traces represent antibody-mediated internalization of virions, while the black trace represents background internalization of virions in the absence of antibody, and the grey solid area is the negative control without inclusion of virus. **C.** To exclude the effects of surface-bound virus, after incubation with virus, THP-1 cells were additionally incubated with 0.05% trypsin for 10 minutes at 37°C just before fixation. Flow cytometry histograms are shown of parallel setups with and without trypsin, using CH31 IgG/HIV-1_BaL_-Tomato immune complexes or negative control anti-RSV Palivizumab/HIV-1_BaL_-Tomato immune complexes.
(TIF)

S2 Fig Erosion of pixels on the brightfield image distinguishes depth of internalization. **A.** THP-1 cells were incubated with CH31 IgG1 and HIV-1_BaL_-Tomato to allow virion internalization. Prior to fixation, the cells were also stained with the surface stain CD14-PE-Cy7 and the nuclear stain DAPI. More than 1400 single, focused cells were acquired using an ImageStream^X^ Mark II (EMD Millipore). AMNIS IDEAS software (v6.1) was used to analyze the images. The intensity of HIV-1_BaL_-Tomato, CD14-PE-Cy7, and DAPI fluorescence was calculated across a defined cell area. At 0 pixels eroded, this area is defined by the entire brightfield image of the cell. The peripheral areas of the cell are excluded from calculation as pixels are eroded from the perimeter of the brightfield image, up to an erosion of 12 pixels. Thus, fluorescence that is on the periphery of the cell is lost as the outer pixels are eroded. The surface stain CD14-PE-Cy7 is preferentially lost compared to the nuclear stain DAPI as pixels are eroded, as shown by a more rapid loss in percent fluorescence intensity compared to the uneroded image. This indicates that erosion of pixels distinguishes internal from surface fluorescence. HIV-1_BaL_-Tomato virion fluorescence is lost at an intermediate rate between the surface and nuclear stains, in line with its assumed endosomal localization, which is intermediate between the nucleus and plasma membrane. **B.** The percentage loss in fluorescence intensity with increasing pixel erosion was graphed for CH31 IgG1, CH31 IgG3, and CH31 mIgA1-associated HIV-1_BaL_-Tomato immune complexes internalized by primary monocytes. Similar fluorescence intensity loss occurs as erosion is increased, indicating that the depth of internalization of immune complexes is similar across antibody isotype/subclass.
(TIF)

S3 Fig Differing internalization phenotypes of THP-1 cells and primary monocytes. **A.** To understand the effect of immune complex size on phagocytosis efficiency in THP-1 cells and primary monocytes, the uptake of ConSgp140-conjugated 1 μm, 0.2 μm, or 40 nm fluorescent beads was analyzed by flow cytometry. Resulting phagocytosis scores from 2 independent experiments are reported. Dashed lines indicate background phagocytosis levels, measured by the mean + 3 standard deviations of relevant negative controls. **B.** To compare the efficiencies of THP-1 cells and primary monocytes for IgG-mediated HIV-1 antigen-conjugated bead phagocytosis, the uptake of immune complexes comprising IgG and ConSgp140-conjugated 1μm fluorescent beads was examined by flow cytometry (N = 3–5 independent experiments). For experiments with primary monocytes, 3 donors were used, with at least 2 replicates for all donors except 1. Negative antibody controls used were non-HIV-1-specific antibodies CH65 IgG1 or Palivizumab IgG1. **C.** To compare the efficiencies of THP-1 cells and primary monocytes for IgG-mediated HIV-1 virion internalization, the uptake of IgG/HIV-1_BaL_-Tomato immune complexes by THP-1 cells or primary monocytes (5 donors, at least 2 replicates for all donors except 1) was examined by flow cytometry (N = 7–9 independent experiments).
(TIF)
