# Supplementary figures and images for: Antibody-Mediated Internalization of Infectious HIV-1 Virions Differs among Antibody Isotypes and Subclasses
Source: PLoS Pathog. 2016 Aug 31;12(8):e1005817. doi: 10.1371/journal.ppat.1005817 (PMC5007037; doi:10.1371/journal.ppat.1005817)

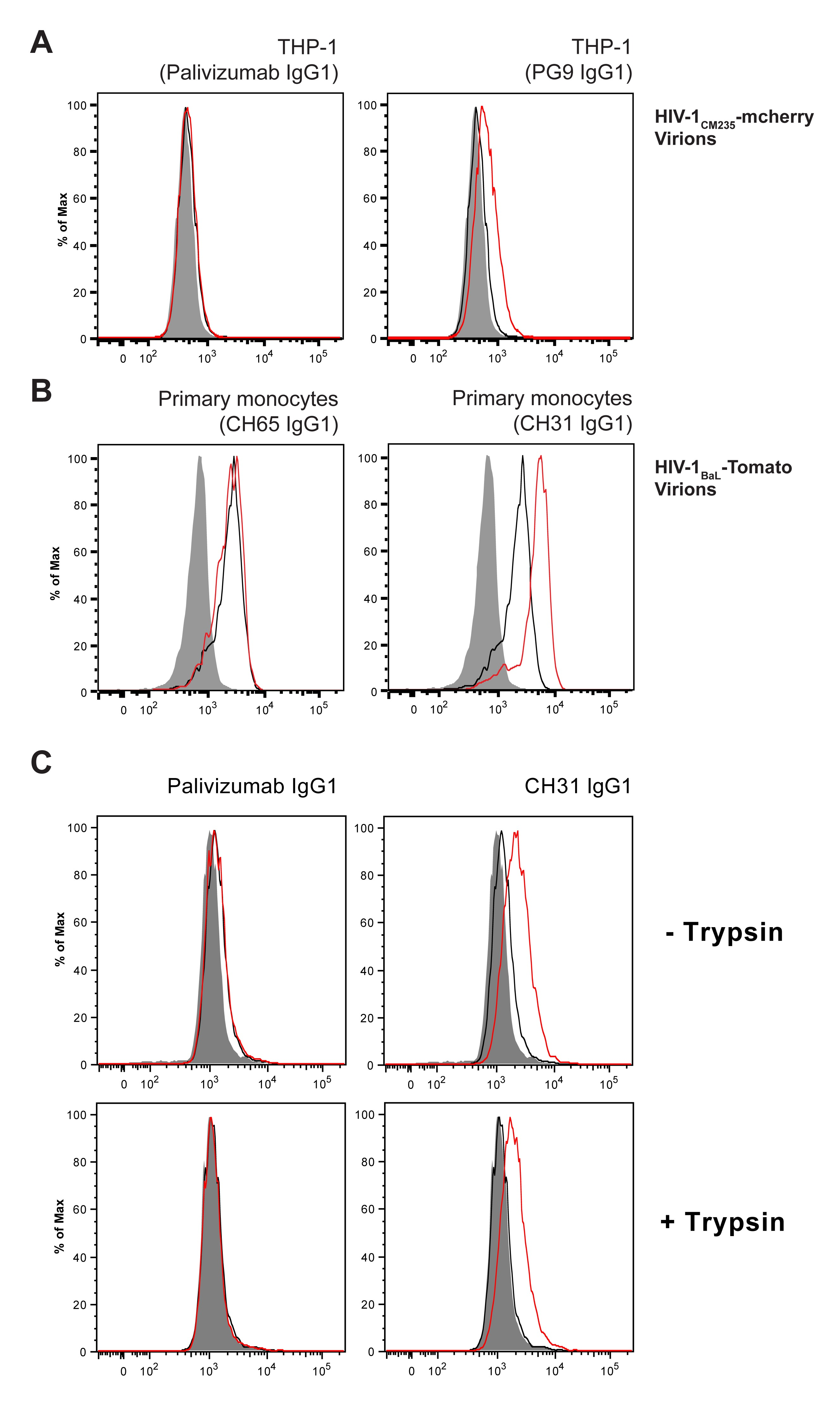

Supplement: S1 Fig — A-B. Internalization of infectious HIV virus was tested by incubating PG9 IgG/HIV-1CM235-mcherry immune complexes with THP-1 cells (A) or by incubating CH31 IgG/HIV-1BaL-Tomato immune complexes with human primary monocytes (B). Representative flow cytometry histograms of independent experiments (N = 2 and N = 8 respectively) are shown. Red traces represent antibody-mediated internalization of virions, while the black trace represents background internalization of virions in the absence of antibody, and the grey solid area is the negative control without inclusion of virus. C. To exclude the effects of surface-bound virus, after incubation with virus, THP-1 cells were additionally incubated with 0.05% trypsin for 10 minutes at 37°C just before fixation. Flow cytometry histograms are shown of parallel setups with and without trypsin, using CH31 IgG/HIV-1BaL-Tomato immune complexes or negative control anti-RSV Palivizumab/HIV-1BaL-Tomato immune complexes. (TIF) [file ppat.1005817.s001.tif]

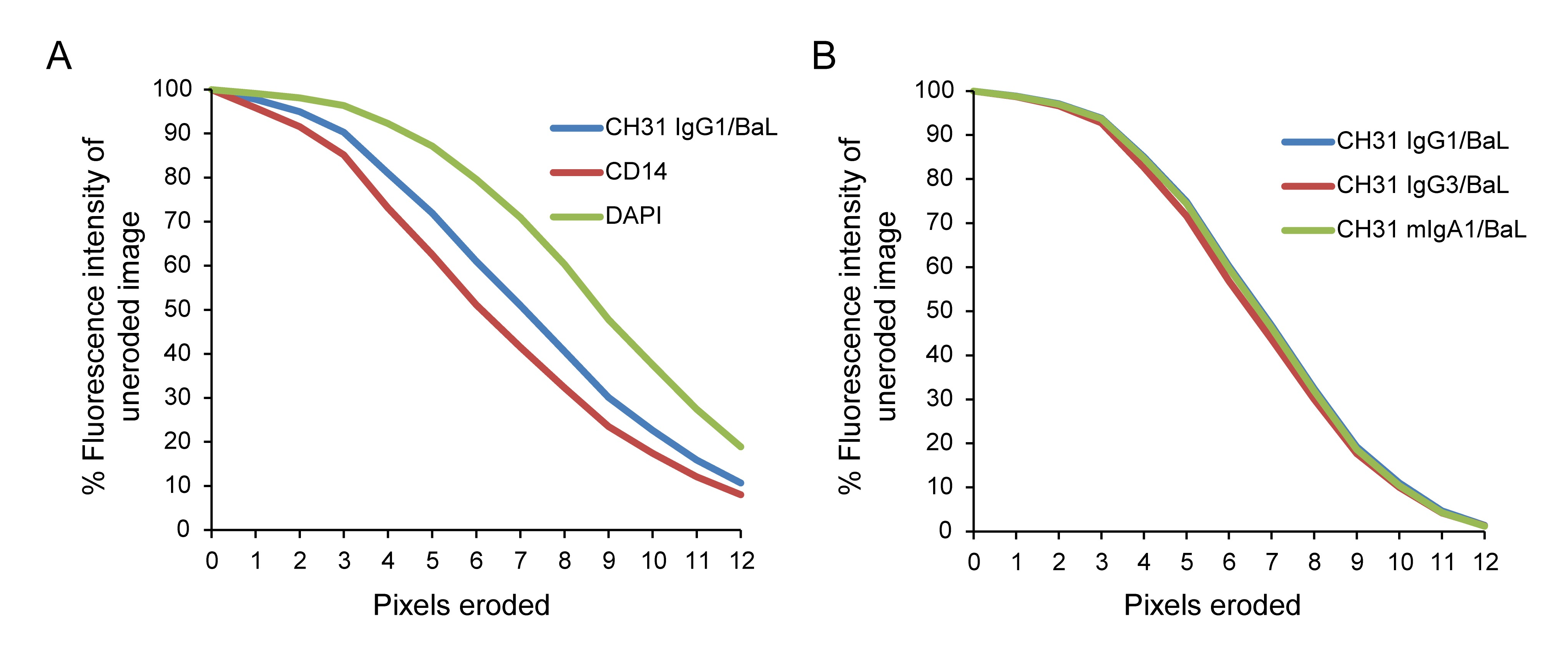

Supplement: S2 Fig — A. THP-1 cells were incubated with CH31 IgG1 and HIV-1BaL-Tomato to allow virion internalization. Prior to fixation, the cells were also stained with the surface stain CD14-PE-Cy7 and the nuclear stain DAPI. More than 1400 single, focused cells were acquired using an ImageStreamX Mark II (EMD Millipore). AMNIS IDEAS software (v6.1) was used to analyze the images. The intensity of HIV-1BaL-Tomato, CD14-PE-Cy7, and DAPI fluorescence was calculated across a defined cell area. At 0 pixels eroded, this area is defined by the entire brightfield image of the cell. The peripheral areas of the cell are excluded from calculation as pixels are eroded from the perimeter of the brightfield image, up to an erosion of 12 pixels. Thus, fluorescence that is on the periphery of the cell is lost as the outer pixels are eroded. The surface stain CD14-PE-Cy7 is preferentially lost compared to the nuclear stain DAPI as pixels are eroded, as shown by a more rapid loss in percent fluorescence intensity compared to the uneroded image. This indicates that erosion of pixels distinguishes internal from surface fluorescence. HIV-1BaL-Tomato virion fluorescence is lost at an intermediate rate between the surface and nuclear stains, in line with its assumed endosomal localization, which is intermediate between the nucleus and plasma membrane. B. The percentage loss in fluorescence intensity with increasing pixel erosion was graphed for CH31 IgG1, CH31 IgG3, and CH31 mIgA1-associated HIV-1BaL-Tomato immune complexes internalized by primary monocytes. Similar fluorescence intensity loss occurs as erosion is increased, indicating that the depth of internalization of immune complexes is similar across antibody isotype/subclass. (TIF) [file ppat.1005817.s002.tif]

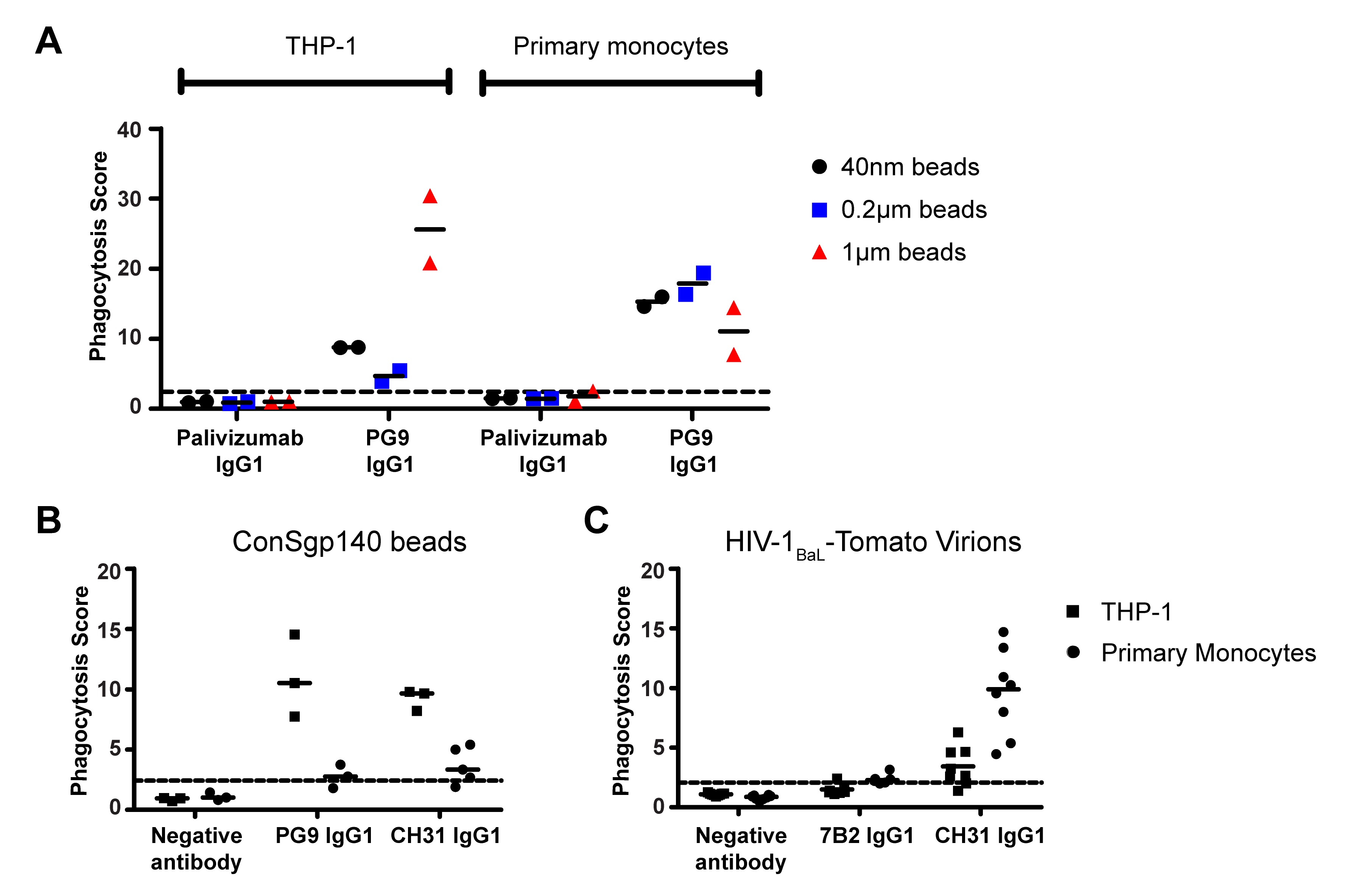

Supplement: S3 Fig — A. To understand the effect of immune complex size on phagocytosis efficiency in THP-1 cells and primary monocytes, the uptake of ConSgp140-conjugated 1 μm, 0.2 μm, or 40 nm fluorescent beads was analyzed by flow cytometry. Resulting phagocytosis scores from 2 independent experiments are reported. Dashed lines indicate background phagocytosis levels, measured by the mean + 3 standard deviations of relevant negative controls. B. To compare the efficiencies of THP-1 cells and primary monocytes for IgG-mediated HIV-1 antigen-conjugated bead phagocytosis, the uptake of immune complexes comprising IgG and ConSgp140-conjugated 1μm fluorescent beads was examined by flow cytometry (N = 3–5 independent experiments). For experiments with primary monocytes, 3 donors were used, with at least 2 replicates for all donors except 1. Negative antibody controls used were non-HIV-1-specific antibodies CH65 IgG1 or Palivizumab IgG1. C. To compare the efficiencies of THP-1 cells and primary monocytes for IgG-mediated HIV-1 virion internalization, the uptake of IgG/HIV-1BaL-Tomato immune complexes by THP-1 cells or primary monocytes (5 donors, at least 2 replicates for all donors except 1) was examined by flow cytometry (N = 7–9 independent experiments). (TIF) [file ppat.1005817.s003.tif]
